# Reactivation of a Retarded Suspension of Ground Granulated Blast-Furnace Slag

**DOI:** 10.3390/ma9030174

**Published:** 2016-03-07

**Authors:** Nick Schneider, Dietmar Stephan

**Affiliations:** Building Material and Construction Chemistry, Technische Universität Berlin, Gustav-Meyer-Allee 25, Berlin 13355, Germany; nick.schneider@tu-berlin.de

**Keywords:** retardation, reactivation, ground granulated blast-furnace slag, d-gluconic acid, NaOH, hydration study

## Abstract

An effective retarded suspension of ground granulated blast-furnace slag (GGBFS) needs a strong activator to reactivate the hydration. In this research study, sodium hydroxide (NaOH) as an alkali activator in two different concentrations (30 and 50 wt.%) was used to overcome the retardation and give the hardened GGBFS the reasonable strength. The study was carried out with a mixture of GGBFS, a solution of 1.0 wt.% d-gluconic acid (C_6_H_12_O_7_) as a retarder in the mixing water and a methyl cellulose as a stabilizer. The reactivation was executed after seven different periods (up to 28 days) after the system was retarded. The following investigations were performed: slump test, measurement of ultrasonic (US) velocity, compressive strength and gross density, thermogravimetry (TG) and scanning electron microscopy (SEM). The analyses of the hardened samples were carried out seven, 28 and 90 days after the reactivation. The result of the study is an effective reactivation of a retarded suspension. In this case, the activator with 50 wt.% NaOH shows a very high performance. The setting time of the reactivated binders is much longer compared to the reference, but, in the longer term, the compressive strength and the progress of the hydration exceed the performance of the reference.

## 1. Introduction

The vision of this research is the development of a plastic inorganic material, which is retarded for at least 28 days and that can be used as a binder after mixing with an activator (two-component system). A possible application of this binder system is a ready to use two-component binder, which is expressible out of a cartridge with two chambers and a static mixer for a quick, simple and safe use. In this study the plastic binder consists of ground granulated blast-furnace slag (GGBFS) and a solution of 1.0 wt.% d-gluconic acid, in respect to the mixing water. The effective retarded binder can be reactivated with sodium hydroxide (NaOH) after different periods of storage.

The retardation in a system with ordinary Portland cement (OPC) occurs by preventing the hydration of the aluminate [1,2,3,4]. Thereby, the dormant period of the hydration process is significantly extended and the formation of calcium silicate hydrate phases (C-S-H-phases) is also prevented [3,5,6,7]. This effect is very probably based on a strong complexation of the calcium with the retarder [5,7,8,9].

The d-gluconic acid, used in this study as retarder, belongs to the group of the carboxylic acids. It is known that the carboxyl groups of a substance can have a possible influence on the plasticization by using them in an alkaline binders [10,11,12]. The effect depends on the electrostatic and steric repulsion of the binder particles [6,11]. Therefore, it is significant to analyze the possible rheological changes when using carboxyl acids. Experiments from Rickert [5] on the slump of concrete with carboxylic acids as the retarder show the plasticizing effect. Similar results are represented by Ludwig *et al.* [9] with saccharose as the retarder. By using nondestructive US-tests it is possible to detect the influence of additives like retarders on the texture of the hydration products [13,14,15]. Retarded binder systems show a late increase of the US-velocity; on the other hand, systems with an accelerator illustrate an early increase [16,17]. An S-shape characteristic of the US-velocity is typical for cementitious systems [16,18]. A low US-velocity and a following strong increase is a good indication for the dormant period of the hydration [17,18]. The onset and offset of the S-shape shows a good correlation with the beginning and the end of the setting [19].

The GGBFS has a high glass content, which can be activated by an increasing pH-value caused by an alkaline activator like NaOH. However at a reaction with water, the GGBFS and the OPC generate similar hydration products, although the GGBFS has a lower reactivity compared to the OPC. On the other hand binders based on GGBFS generally develop a denser microstructure with a finer structure of C-S-H-phases, due to the absence of portlandite [20,21,22,23].

The results of different studies [5,9,24,25,26] show in the long term a neutral to possible positive influence on the strength-development of binder systems by using retarders. However, a gain in strength only occurs with appropriate retarder concentrations and after long hydration times [5,9,25]. A negative aspect is the possible lack of strength at the beginning of hydration. This occurred in experiments by Rickert [5] and Luke *et al.* [24] who used saccharose at concentrations higher than 0.1% by weight of cement. The demolding of Rickerts samples was only possible after several weeks. Similar results are reported by Ludwig *et al.* [9] and Lieber *et al.* [25], whereupon Ludwig explains the effect by the retarded alite.

The reactivation of retarded binders is known from the fresh concrete recycling process, where recycling additives are used to retard the hydration process by several days. In this case, the retarded mass is mixed with fresh concrete to overcome the retardation and initiate the hydration again [5]. According to Langenfeld *et al.* [2], the calcium complexes with the retarder are destroyed by the increase of the pH-value and the setting can occur. With the alkaline activation, the pH-value is not the only influencing factor, but also the composition and the concentration of the activator has an effect [27,28,29,30,31,32,33,34]. The results of some studies [32,33,35,36,37] show an increase in strength and a higher degree of hydration of the binder due to an increase of the activator concentration. Generally, the main products of the hydration of the GGBFS are C-S-H-phases, but their microstructure depends strongly on the available basicity [38,39,40]. The structure of these phases is similar to tobermorite or also jennite [38,39,41,42]. Hydrotalcite is another product which is formed when a relevant high content of magnesium oxide (MgO) [42,43,44] or a high activator concentration is present [41].

Similar multi component systems with different binders, but with the same idea—the regulation of the hydration process—were also studied by Pang *et al.* [7] and Brothers *et al.* [45].

## 2. Materials and Methods

**Figure 1:**
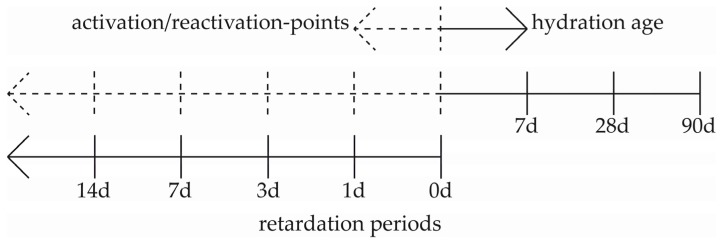
Timescale of the retardation periods (0–28 days), the points of activation/reactivation (Ac1, Ac2) and the ages of hydration (7–90 days).

The retarded suspension consists of GGBFS, a solution of 1.0 wt.% d-gluconic acid (1%R) and methyl cellulose (MC). The MC has a share of 0.1 wt.% in respect of the whole retarded mass. This combination of admixtures leads to an effective retardation and stabilization over the different periods of time (Figure 1). Some factors vary in this study. These are the retardation periods, the points of reactivation of the retarded suspension, the activator concentration and the hydration time. Figure 1 is a schematic illustration of these factors with their applied effective period during the tests of the hydration study. This leads to a multilayer series of experiments and results of the analyses.

The chemical composition and some physical properties of the GGBFS are shown in Table 1. The GGBFS has a basicity (CaO/SiO_2_) of 1.21, which is close to the average of German GGBFS (*cf.* [46]). The original d-gluconic acid has a concentration of 50 wt.%, a density of 1.24 kg/dm^3^ and a pH-value of 1.82. The MC has a density of 1.39 kg/dm^3^ and a maximum adjustable viscosity of 24,600 mPa·s. The activating solutions were produced by dissolving NaOH-pellets (purity of over 99 wt.%) in demineralized water with a final concentration of 30 and 50 wt.% and a resulting pH-value of over 14.

For the testing, a mixture of 1 dm^3^ for each retarded period was produced. The mixing was performed with a mortar mixer (type 205 from TESTING Bluhm & Feuerherdt GmbH, Berlin, Germany) at a speed of 140 rpm. The mixing of the retarded mass (GGBFS, retarder solution, MC) consists of homogenization of the dry materials, mixing with the retarder solution, a break and repeated mixing, each section for 1 min. The water-binder ratio (w/b-ratio) for the mixture was 0.30. Each retarded mass was stored in hermetically sealed containers (volume of 3.6 dm^3^) at 20 °C until the respective use.

The activation of the retarded samples in the desired ages were done after 1 min of homogenization followed by mixing with 9.9 wt.% of the activator with respect to the whole activated mass (mass with retarder and activator) for 1 min. Due to the addition of the activator, the w/b-ratios increased for the mixture with 30 wt.% NaOH (Ac1) to 0.40 and for 50 wt.% NaOH (Ac2) to 0.37. In order to interpret the results more accurately, the reference for each activator was also prepared without the retarder but with the same w/b-ratio and the materials of GGBFS, MC, water and the respective activator.

### 2.1. Slump Test and the Measurement of US-Velocity

The measurement of the slump flow after the storage of the retarded samples was performed according to DIN EN 1015-3 [47] to describe the rheological properties of the mixtures. In deviating from the standard, a single slump flow was determined and the dimensions were measured before and 5 min after the strokes. This pursues the purpose to record the thixotropy and the stability of the mixture. The tests were carried out with an automatic flow table (from TESTING Bluhm & Feuerherdt GmbH, Berlin, Germany) at 20 °C and 65% relative humidity.

A fixed part of 0.6 dm^3^ from the retarded mass was mixed with the activator. The point in time at which the activator is added is regarded as actual beginning of the hydration (Figure 1).

The measurements of the US-velocity (Ultratest IP-8 from UltraTest GmbH, Achim, Germany) were carried out in a double determination to illustrate the hydration process and its parts of the beginning and the end of setting. The reactivated suspension was filled into the measuring chamber within 2 min under constant manual compaction to eliminate air bubbles, which influence the measurement. The samples were covered with a plastic film to protect from drying-out. The measurement was carried out for seven days after the activation and the data signal was generated every minute. The incorrect data points were removed from the analysis.

### 2.2. Compressive Strength and Gross Density

The samples for testing the compressive strength and the gross density were produced from the activated suspensions according to DIN EN 196-1 [48]. In deviating from the standard and with the aim of practical representation, another size of the samples (2 × 2 × 2 cm^3^), other periods of demolding (3–7 days), another kind of storage (20 °C and 100% relative humidity) and no compaction were used. The samples were stored until the tests were performed after seven, 28 and 90 days of hydration (Figure 1).

The compressive strength was determined by a compression test machine (type 2060 from Toni Technik Baustoffprüfsysteme GmbH, Berlin, Germany). The size and the weight of the samples were recorded digitally for the calculation of the gross density. Before the digital measurement, every sample was polished to remove the irregular top surface.

### 2.3. Thermogravimetry (TG) and SEM

The samples for the analyses by TG and SEM were taken from the remaining fragments of the compressive strength test. The samples were dried in a vacuum freeze-drying device (Alpha 1-4 LSC from Martin Christ Gefriertrocknungsanlagen GmbH, Osterode, Germany), at a pressure of approximate 1 mbar.

The TG measurements (TG 209 F3 Tarsus from NETZSCH-Gerätebau GmbH, Selb, Germany) were performed in the range from ambient temperature up to 1000 °C (10 K/min with a purging rate of 20 mL/min with nitrogen) to quantify hydration products. The dried samples were ground in a vibration disc mill to approximate 6900 cm^2^/g according to Blaine. The results were evaluated by the Marsh procedure.

The pictures by SEM were taken at 20 kV with a secondary electron detector and a pressure of approximate 1 × 10^−6^ mbar. For this purpose, a device (S-4000 from Hitachi Ltd., Tokyo, Japan) with a cold field emission cathode was used. The samples were prepared using conductive silver and sputtered by gold prior to the measurement.

## 3. Results and Discussion

The results show a general feasibility of the reactivation of the GGBFS suspension after a long period of retardation. Compared to Ac1, Ac2 is more effective, decisively due to its higher concentration. The results of Ac2 are an earlier setting, higher absolute compressive strength and a denser structure with more hydration products.

### 3.1. Slump Test

The slump flows of the retarded mixtures after different periods of storage are uniform. The average of these mixtures is 10.5 cm before and 19 cm after the strokes (deviations are insignificant). The reference without a retarder has a lower slump flow, 10 cm before and 16 cm after the strokes. However, all mixtures showed a thixotropic behavior. These results illustrate that at least some of the retarder adsorb onto the binder (the reversal of the electric charge on the binder particles), which was detected already in a similar way by Rickert [5] and Ludwig *et al.* [9]. Some other researchers [6,10] observed this property of the plasticizing effect of the carboxyl group as well. No separations occurred, which confirms the effectiveness of the MC with a w/b-ratio of 0.30. A reactivated mixture has a much higher slump flow (approximate 30 cm) as the mixture without activator, due to the higher w/b-ratio of 0.37 (Ac2) and 0.40 (Ac1).

### 3.2. Measurement of US-Velocity

**Figure 2:**
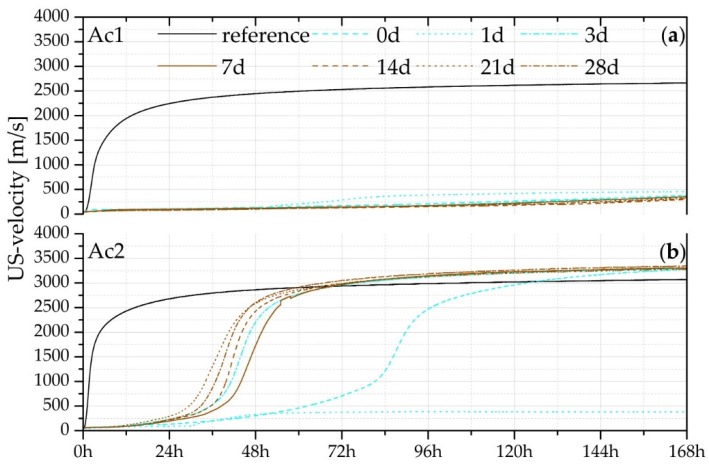
Average of US-velocity measurement of the references (without retarder) and the reactivated mixtures with (**a**) Ac1, and (**b**) Ac2 after seven different periods of retardation with 1%R.

Figure 2 shows the results of the US-measurements of the reactivated mixtures with Ac1, Ac2 and their references. The references present a quick hydration course almost without a dormant period. The reactivations with Ac1 do not overcome the retardation completely and do not reach the setting within seven days, which is presented in Figure 2a. However, in comparison to non-reactivated mixtures, the results in Figure 2a have a constant increase of the US-velocity. Further measurements with GGBFS and retarder solution of 1.0 wt.% illustrate that no change in the US-velocity occurs for up to seven days. This supports the finding that the acceleration period of Ac1 reactivations was not overcome within seven days but the hydration is still in progress, therefore the mixtures are not absolutely retarded. Potentially, the activator has not activated the binder grains sufficiently, so that the setting does not take place due to the growth of the C-S-H-phases.

In contrast to the reactivations with Ac1, Ac2 has a significantly stronger influence on the retarded mixtures (compare a and b of Figure 2). All samples show overcoming of the retardation within seven days. A general exception by all reactivations is the sample with a retardation of one day; this was a mismeasurement of a wrong reactivation. These results are shown, to illustrate the effectiveness of the retarder. The reactivated samples from three to 28 days reach their setting in a period between 28 and 38 h after the addition of Ac2. Under the influence of the retarder, all reactivated systems with Ac2 show an extension of the dormant period of at least 24 h. After this period, they reach the beginning of the setting and then the maximum US-velocity with forming an S-shape course of the hydration (*cf.* [16,17,18,19]).

Table 2 shows the maximum US-velocity of the reactivations with Ac2 and their points in time at which 10, 50 and 90% of the maximum were reached. Due to the similar maximum US-velocity of all reactivated mixtures (average of 3300 m/s), it becomes clear that the US-velocity of the 21 and 28 days old samples is faster than the others. In comparison with this, the reactivation after day 0 is much slower. This behavior and the higher US-velocity in comparison to the references are based on the effect of the retarder (*cf.* [13]). The question about the lower reactivation by a shortly retarded sample (day 0) is still open. Perhaps the combined addition of retarder and activator blocks the single reaction of each agent. Thus, it is not possible for the retarder to adsorb totally on the binder grains, because the basic activator is also in the solution.

From the work of Walz *et al.* [49] and Richartz [50], it is known that the retarder use in a long term can increase the hydration and therefore also the compressive strength. Due to the approximate 7.5% higher maximum US-velocity of Ac2 systems in comparison to their reference, it can be assumed that the retarder has also a positive influence on the compressive strength.

### 3.3. Compressive Strength and Gross Density

**Figure 3:**
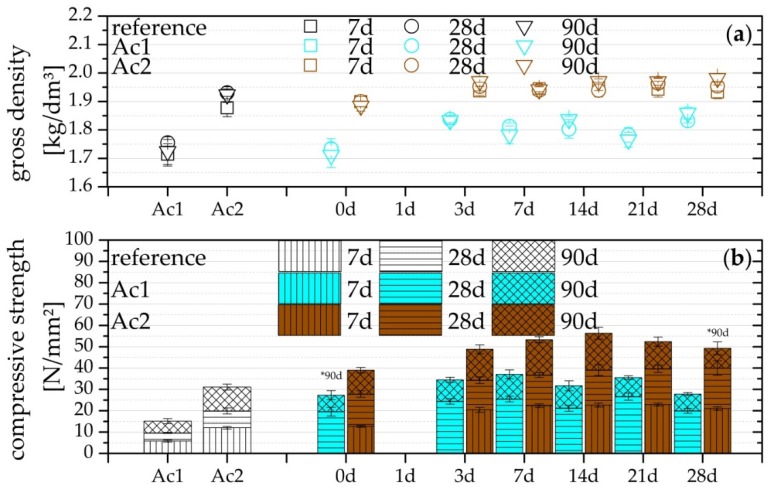
(**a**) Gross density and (**b**) compressive strength after seven, 28 and 90 days of hydration, of the reference (without retarder) and the reactivated mixtures with NaOH after seven different periods of retardation with 1%R.

Generally, it is possible to reach a higher compressive strength with the use of the retarder, which is observable from the result in Figure 3. Indeed, the effect is detectable mostly after the tests of the compressive strength after 28 days of hydration. The reactivated samples from three to 28 days with Ac2 show the positive effect of the retarder already after seven days of hydration. Similar results were found in [4,5,9,25,26,49,50]. Richartz [50] has ascribed the increase of the compressive strength to the development of long-fiber C-S-H-phases, due to the increase of the dormant period.

The results in Figure 3 are affected basically by three factors, the retarder, the different concentrations of the activator, and the various w/b-ratio of both systems. The various w/b-ratios are a consequence of the different activator concentrations and cannot be considered separately. However, the higher compressive strength of systems with Ac2 in comparison to Ac1 cannot only be based on the lower w/b-ratio (approximate 0.03). Thereby, the main reason is the higher concentration and also, for the reactivated samples, a quicker setting, shown by the results of the US-velocity. Therefore, samples with a quicker setting have more time to form hydration products, whereby a higher compressive strength can be achieved. This assumption is supported by further analyses of the compressive strength from mixtures only with GGBFS and w/b-ratios of 0.30 and 0.35. The mixture with a w/b-ratio of 0.30 has a compressive strength of 27.2 N/mm^2^ after 28 days and 39.8 N/mm^2^ after 90 days of the hydration (standard deviation of 0.6 and 0.8 N/mm^2^). The mixture with the higher w/b-ratio has nearly the same compressive strength, 26.5 N/mm^2^ after 28 days and 41.8 N/mm^2^ after 90 days of the hydration (standard deviation of 1.4 and 2.5 N/mm^2^).

The results of the gross density and the compressive strength are rather similar about the different retardation periods and for each activator, with the exception of the reactivation after 0 day. The results in Figure 3a are the same or above their reference. This shows the plasticizing influence of the retarder which was already analyzed with the slump flow (*cf.* [5,6,9,10]) and the increased degree of hydration products.

The average of the results from the compressive strength of 0 day Ac1 and 28 days Ac2 of the test after 90 days of hydration was generated with less than five individual results (show in Figure 3 with the mark of *90d). Because of the high divergence, only four results were used. The samples reactivated after one day of retardation did not reach their setting even after 90 days of hydration. This represents the effectiveness of the retarder and also the possible failure of the reactivation.

The reactivations with Ac1 have no setting within seven days; therefore, no compressive strength is presented. However, all systems with retarder reached a higher compressive strength after the 28 days of hydration with at least 20 N/mm^2^ more than their reference. The relative increase in strength of the reference of Ac1 and Ac2 from the 28 to the 90 days of hydration is above their retarded systems, but the average increase of approximate 30% from both retarded systems is almost the same. The difference of the relative increase in the compressive strength of the both systems with different activators is significant up to the 28 days, after the addition of the activator. After the 28 days of hydration, the difference is not present in the relative condition of the results but only in the absolute condition (Figure 2b). GGBFS is well-known for the high increase of the compressive strength after the 28 days of hydration [21,46].

Based on the results, it is assumed that the high activator concentration dissolves more slag resulting in the quick setting, whereby a considerable amount of strength forming phases are produced. This is the most likely reason for the difference of the systems of Ac1 and Ac2 because the low disparity of the w/b-ratio (0.03) cannot be the only reason for the difference of at least 10 N/mm^2^. From the results of the compressive strength, it can be concluded that the overcoming of the probable complexes of calcium and retarder is reached faster if using an activator with a higher concentration. The outcome of this is a higher absolute compressive strength at all times at which the retarded samples were reactivated.

### 3.4. TG

**Figure 4:**
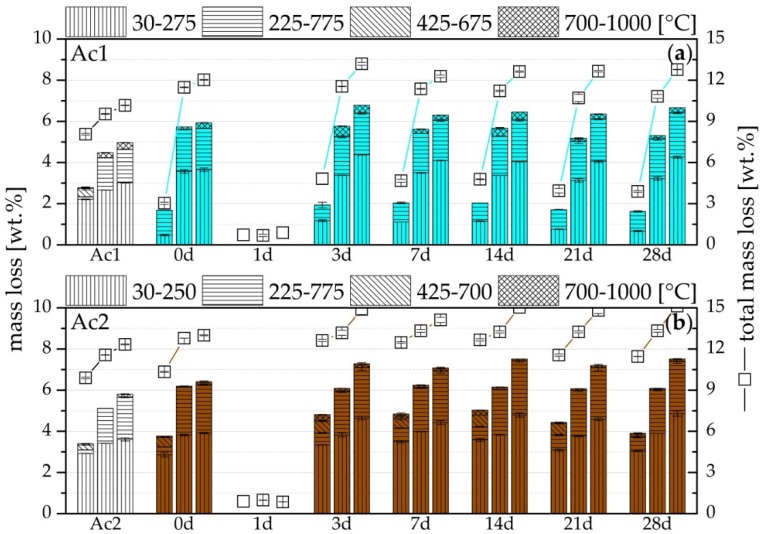
Mass loss by TG of (**a**) Ac1 and (**b**) Ac2 after seven, 28 and 90 days (from left to right per test) after the beginning of the hydration of the references (without retarder) and the reactivated mixtures after seven different periods of retardation with 1%R.

Figure 4 shows the generally appearing increase of the hydration products from seven to 90 days of hydration. This result is independent from the time at which the retarded suspension is reactivated but it is not independent from the used activator. Generally, it is possible to overcome the retarded state with both activators, however the samples with Ac2 are quicker, more effective and they contain more C-S-H-phases. The C-S-H-phases are detectable from the difference between the total mass loss and the summation of the mass losses from the single temperature ranges from 30 to 1000 °C (Figure 4). The highest difference between both activators exists after seven days of hydration. The analysis by the Marsh procedure can separate several peaks of the mass loss and also a main part of the linear mass losses, those of the C-S-H-phases. Therefore, the difference between the total mass loss and the summation of the single mass losses from 30 to 1000 °C is mainly the decompositions of C-S-H-phases. However, the remaining linear part from the mass losses of the C-S-H-phases dehydrates at the peaks of the single illustrated temperature ranges until at least 700 °C. Hence, this part is not separable. The results also show that the total mass loss of the Ac2 systems is always higher than those of the Ac1 systems, whereby the higher degree of hydration of the systems with Ac2 are illustrated. Therefore, a strong correlation exists between the results of the TG and the compressive strength. The positive influence of the retarder becomes visible at the latest by the systems of Ac1 after 28 days of hydration. Within this time, the reactivated systems have formed the same or a higher amount of hydration products compared to their reference without retarder.

The results of the systems with Ac1 after seven days of hydration show detectable products (Figure 4a), but the samples have no setting. All samples with a retardation of one day have also no setting and showed no hydration products. The faulty reactivation after one day of retardation shows a fully retarded state. This state is not present at the systems with Ac1 after seven days of hydration, but the common characteristic of both states is the absence of any compressive strength. The TG results of the systems with Ac1 until seven days of hydration correlate with the characteristic of the US courses in Figure 2a. The TG analysis illustrates only a little part of strength giving phases, which is the reason that the setting was not present and the samples did not achieve any compressive strength after seven days of hydration.

**Figure 5:**
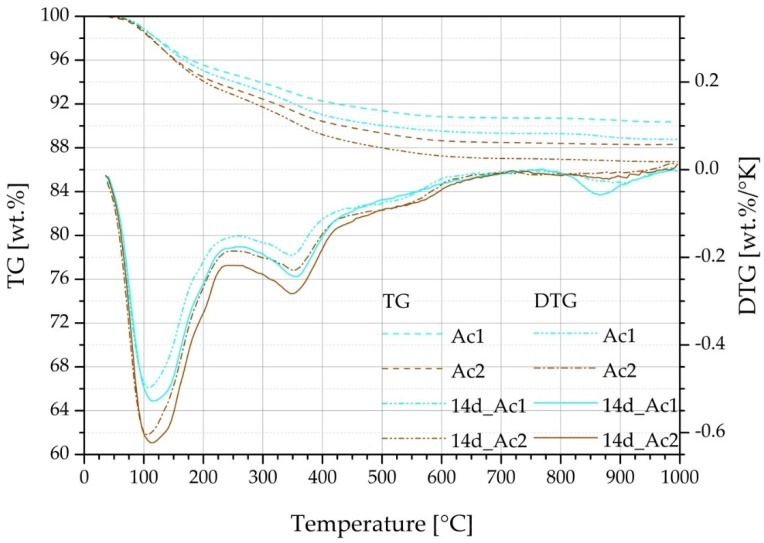
TG and DTG of the references (Ac1 and Ac2, without retarder) and its reactivations after 14 days of retardation with 1%R after a hydration period of 28 days.

The products of the temperature range between 30 and 250/275 °C can be assigned mostly to C-S-H-phases (*cf.* [40,43]). The compounds with magnesium mainly hydrotalcite are the dominant products of the range of 225–775 °C (*cf.* [40,43]). It should be noted that the increase of this mass loss occurs mainly between seven and 28 days of hydration (Figure 4). This is independent from the point in time of reactivation, the retarder and the concentration of the activator. The products of the temperature range 425–675/700 °C exist temporarily and only after the hydration of seven days and mostly with the Ac2 systems. It can be assumed that these products are converted to more stable compounds during the ongoing hydration. Comparing the products of the systems with Ac1 and Ac2 in the temperature range between 700 and 1000 °C, only the products of Ac1 have a clear peak (Figure 5). Due to the lower hydration in comparison to the Ac2 systems, the development of the formed products is detectable only from the 28 days of hydration. The resulted products can be assigned to carbonates (*cf.* [51]).

Gruskovnjak *et al.* [40] and Ben Haha *et al.* [43] have analyzed similar hydration products in their researches with alkali activated GGBFS.

### 3.5. SEM

**Figure 6:**
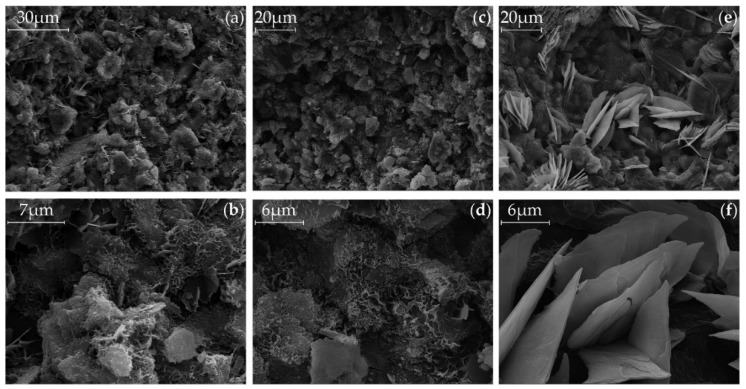
SEM pictures with different magnifications after a hydration period of 28 days, (**a**) and (**b**) of the reference of Ac1, (**c**) and (**d**) of the surface, (**e**) and (**f**) of pore of the reactivated mixtures with Ac1 after 14 days of retardation with 1%R.

**Figure 7:**
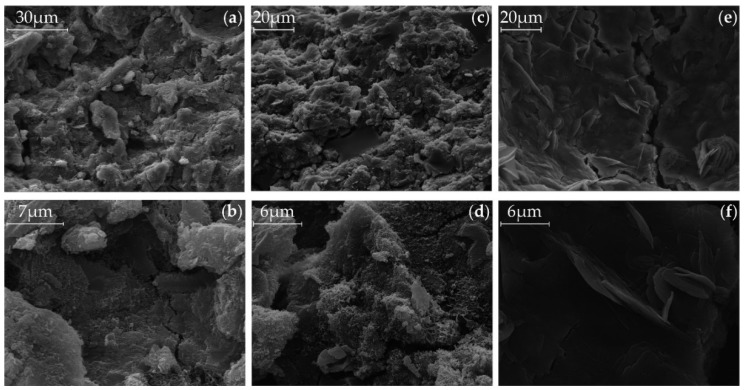
SEM pictures with different magnifications after a hydration period of 28 days, (**a**) and (**b**) of the reference of Ac2, (**c**) and (**d**) of the surface, (**e**) and (**f**) of pore of the reactivated mixtures with Ac2 after 14 days of retardation with 1%R.

**Figure 8:**
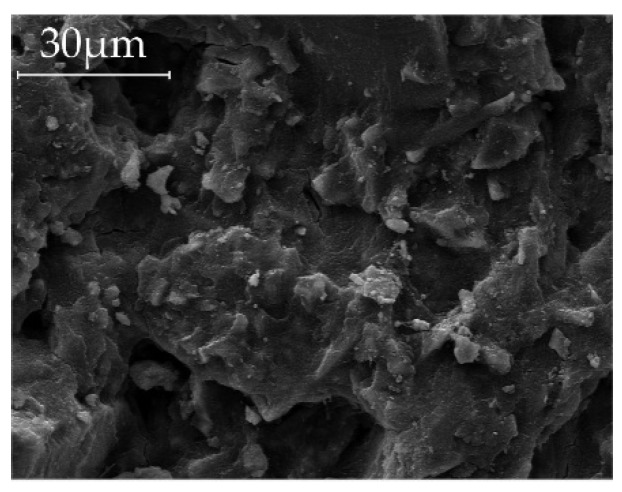
SEM picture after a hydration period of 28 days, of a mixture with GGBFS and demineralized water (without activator).

The Figure 6 (Ac1) and Figure 7 (Ac2) show the microstructure of the activated samples. The SEM pictures (a) and (b) show the microstructure of the references after 28 days of hydration. Hereby, the samples with Ac2 seem to have a denser microstructure. However, the SEM pictures (c) and (d) illustrate the state of the microstructures after reactivation with a previous retardation of 14 days. In the SEM pictures (e) and (f) the content in the pores after reactivation is shown. Generally, the pictures represent the reactivated structure after 14 days of the retardation, which are also representative for other dosing points of the activators. This method also illustrates a different microstructure between both activators, with and without retarder. The system with Ac1 shows basically a porous, grid-like and in some parts hexagonal structures, with lamellar-shaped hydration products in the pores. In comparison to this porous structure, Figure 8 shows a compact structure of a mixture with GGBFS and demineralized water, without activator, after a hydration time of 28 days. It can be assumed that the general porous structure in Figure 6 and Figure 7 is formed as a result of the activator. Ben Haha *et al.* [43] figured out that the porous structure is caused by the quick reaction of the activator. However, in this study, it is obvious that the duration of the acceleration period is responsible for the porosity, because the long-lasting hydration of the systems with Ac1 (Figure 2a) results in no denser structure, compared to their references. The quick reaction of the activator increases the speed of some reactivated system with Ac2 to about 60% of the maximum US-velocity within 12 h.

By comparing the references ((a) and (b)) and the reactivated samples ((c) and (d)) in Figure 6 and Figure 7, it is not clear which structural changes are responsible for the big difference in the compressive strength (Figure 3b). The SEM pictures show no obvious difference in the microstructure. On the other hand, this means that there is no unfavorable effect on the compressive strength by that grid-like structure.

The different characteristic structures in the pores of the reactivated systems are probably based on the different porosities of the samples. Bulky structures, like calcium hydroxide, can be formed in free spaces. An analysis of a bulky pore structure of a sample with Ac1 by using the energy dispersive X-ray spectroscopy (EDX) with point measurement has mostly identified the element calcium. Because of this result and the lamellar-shaped structures in Figure 6e,f, it could be assumed that this is calcium hydroxide. However, this is not possible, because calcium hydroxide does not belong to the general existing phases in an activated GGBFS system (*cf.* [40,43]). In addition, the TG analysis shows no mass losses which corresponds to calcium hydroxide. Another EDX analysis on some points of a grid-like structure show that the structure mainly consists of the elements calcium, aluminum, silicon, sodium and magnesium. This was checked at other reactivated samples. Due to these results, the C-S-H-phases can be confirmed as one of the strength giving phases of reactivated systems. It is still unclear which compounds are formed by the remaining elements and whether these compounds are variable within the progress of hydration or not. Analyses with X-ray diffraction (XRD) could clarify where the high content of magnesium from the GGBFS is bound and whether hydrotalcite arises due to the high content of magnesium.

## 4. Conclusions

The results confirm the feasibility to reactivate the hydration of a 28 days old retarded suspension of GGBFS with NaOH. The analyses have proved that the setting and hardening of the reactivated systems can be controlled by the concentration of the activator. In detail, all retarded suspensions have shown the following characteristics by a reactivation with 50 wt.% compared to 30 wt.% NaOH:

- Setting within 48 h after the addition of the activator (from a retardation of three days)
- Compressive strength after seven days already exceeds the reference
- Formation of more hydration products than the reference, especially C-S-H-phases
- Formation of a denser structure

The influence of the retarder has a positive effect on the results of the slump flow, the gross density, the compressive strength and the formation of the hydration products. In the hydration, the reactivated systems have preponderant advantages over their references. The only disadvantage is the later setting.

The following characteristics are detected by the reactivations of retarded suspensions:

- The beginning of setting is extended, due to the overcoming of the retarded state.
- Higher activator concentration causes a higher absolute compressive strength (also correct for non-retarded systems) and a quicker setting.
- Activator concentration has no effect on the relative increase of the compressive strength from the 28 days of the hydration (also correct for non-retarded systems).

The results show that the activator concentration has the main influence on the setting time whereby the beginning and the duration of the growth of C-S-H-phases is also controlled. Furthermore, the results represent that the texturing of the phases are regulated by the duration of the acceleration period.

## Figures and Tables

**Table 1:** Chemical composition and physical properties of ground granulated blast-furnace slag (GGBFS).

Chemical Composition	GGBFS
SiO_2_	35.6 wt.%
Al_2_O_3_	10.6 wt.%
Fe_2_O_3_	0.7 wt.%
MgO	7.4 wt.%
CaO	43.2 wt.%
Na_2_O	0.2 wt.%
K_2_O	0.4 wt.%
TiO_2_	0.7 wt.%
MnO	0.2 wt.%
**Physical Properties**	
Density	2.92 kg/dm^3^
Specific surface area (Blaine)	3218 cm^2^/g
Water demand (Puntke)	19.1 wt.%
RRSB-Distribution x_0_/n	20.7/1.7

RRSB: Rosin, Rammler, Sperling and Bennet.

**Table 2:** The maximum of the US-velocity at 168 h of the reference (without retarder) and the reactivated mixtures with Ac2 and the points in time when 10%, 50% and 90% of the maximum US-velocity were reached.

Samples of US-Velocity	Reference	0 Day	3 Days	7 Days	14 Days	21 Days	28 Days
US-velocity at 168 h (m/s)	3071	3278	3292	3299	3313	3279	3340
10% of max. at time (h)	0.90	50.18	30.45	33.58	30.78	25.53	29.20
50% of max. at time (h)	2.50	87.16	44.48	47.50	42.48	38.08	40.15
90% of max. at time (h)	26.65	119.12	72.52	71.76	71.28	67.12	67.45
